# Cost‐effectiveness analysis of sintilimab plus IBI305 versus sorafenib for unresectable hepatic cell carcinoma in China

**DOI:** 10.1002/cam4.5724

**Published:** 2023-07-11

**Authors:** Zhe Xu, Zhuo‐miao Ye, Yu‐kai Tang, Dong‐feng Deng, Qin Zhou, Man Fang, Ying‐ying Zhang, Xiang‐ping Li

**Affiliations:** ^1^ Department of Pharmacy Xiangya Hospital Central South University Changsha Hunan China; ^2^ Department of Oncology Xiangya Hospital Central South University Changsha Hunan China

**Keywords:** cost‐effectiveness, hepatocellular carcinoma, Markov model, sintilimab, sorafenib

## Abstract

**Background:**

Sintilimab combined with IBI305 treatment regimen had potential clinical benefits than sorafenib in the first‐line treatment of patients with unresectable hepatic cell carcinoma (HCC). However, whether sintilimab plus IBI305 has economic benefits in China remains unclear.

**Methods:**

From the perspective of Chinese payers, we used the Markov model to simulate patients with HCC receiving treatment with sintilimab plus IBI305 and sorafenib. The transition probability between health states was estimated using the parametric survival model, and the cumulative medical costs and utility of the two treatment methods were estimated. Considering the incremental cost‐effectiveness ratios (ICERs) as the evaluation index, sensitivity analyses were performed to explore the impact of uncertainty on the results.

**Results:**

Compared to sorafenib, sintilimab plus IBI305 generated an additional $17552.17 and 0.33 quality‐adjusted life years, resulting in an ICER of $52817.89. The analysis outcomes were most sensitive to the total cost of sintilimab plus IBI305. With a willingness‐to‐pay threshold of $38,334, sintilimab plus IBI305 showed a 1.28% probability of being cost‐effective. The total cost of sintilimab plus IBI305 should be reduced by at least 31.9% to be accepted by Chinese payers.

**Conclusions:**

Regardless of whether the price of sintilimab plus IBI305 and sorafenib is covered by Medicare, sintilimab plus IBI305 is unlikely to be cost‐effective for first‐line treatment of patients with unresectable HCC.

## 1 INTRODUCTION

Liver cancer, the sixth deadliest form of cancer in the United States, places a huge economic burden on American society.^1^ More than three quarters of liver cancers are hepatocellular carcinomas (HCC), and five‐year survival rate for patients with advanced HCC is <20%.^2^ Surgical resection is highly effective in patients with early‐stage HCC; however, advanced HCC is typically unresectable.^2^ In addition, conventional treatments, such as transarterial chemoembolization and radiofrequency ablation, have limited success in patients with advanced HCC. Therefore, targeted tyrosine kinase inhibitor (TKI)‐based therapy has become the mainstay of treatment for these patients. TKI‐ regimen has been used for over a decade; however, it is associated with only a modest improvement in the overall survival (OS) of patients with HCC of approximately 3 months.^3^, ^4^ In addition, chronic hepatitis B/C virus infection, a common risk factor for HCC, promotes HCC development through programmed cell death protein 1 (PD‐1)/PD‐ligand 1 (PD‐L1)‐mediated suppressive tumor microenvironment.^5^ Immune checkpoint inhibitors (ICIs) can selectively block the PD‐1/PD‐L1 signaling pathway to prevent T‐cell inactivation, thereby improving the ability of the patient's own immune cells to recognize and kill tumor antigens. Therefore, this novel therapeutic approach may overcome the current dilemma of limited treatment options for advanced HCC. Several large randomized controlled trials (RCTs) have reported good clinical benefit from the use of ICIs alone in the treatment of patients with advanced HCC.^6^, ^7^ Treatment modalities such as atezolizumab plus bevacizumab, a combination of ICI and vascular endothelial growth factor inhibitor, further improved outcomes with significantly better OS and progression‐free survival (PFS) than sorafenib.^8^, ^9^ The long‐term survival benefit of atezolizumab plus bevacizumab was further confirmed in a recently published updated analysis of efficacy and safety.^10^ The recent ORIENT‐32 clinical trial reported the clinical benefit of sintilimab plus IBI305 (bevacizumab analog) compared to sorafenib for treating advanced HCC.^11^ The results showed that sintilimab plus IBI305 significantly increased OS and PFS in patients with HCC than sorafenib monotherapy, and the combination of sintilimab plus IBI305 could be an attractive first‐line option for the treatment of unresectable HCC.

Sorafenib was included in medical payments by the National Healthcare Security Administration of China in 2017. The price of sorafenib was reduced from tens of thousands of RMB per box to thousands of RMB per box, which greatly reduced the economic burden on the patients. However, the financial burden of overall care for patients with cancer remains poor, and the high price of oncology drugs limits their use. Therefore, medical institutions must consider whether the expected cost and results of novel cancer combination therapies are reasonable. Although several cost‐effectiveness studies have compared sorafenib with other therapies for advanced HCC, no studies have revealed that IBI305 plus sintilimab is more cost‐effective than sorafenib.^12^, ^13^ Therefore, based on the latest clinical trial data, drug pricing, and guidelines, we developed a Markov model to evaluate whether sintilimab and IBI305 are cost‐effective, which could provide a reference basis for future healthcare policy changes.

## 2 METHODS

### 2.1 Population

The basic medical data used in this economic evaluation include a large multi‐center RCT (ORIENT‐32). We focused on phase III of the ORIENT‐32. The recruited patients were clinically or pathologically diagnosed with locally unresectable or metastatic HCC. This study included 571 patients and included the experimental group (380 patients) that received sintilimab plus IBI305 combination therapy and a control group (191 patients) that received sorafenib monotherapy. There were no statistical differences in the demographic characteristics of the patients in the experimental and control groups.

### 2.2 Model's structure

**FIGURE 1:**
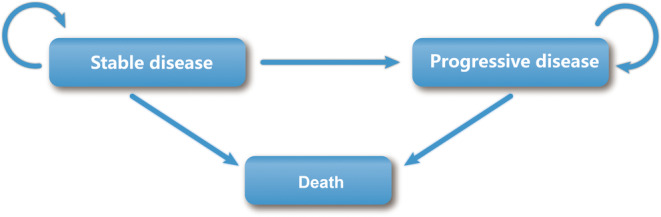
Markov state transition model.

Our analysis included 571 patients who were enrolled in the ORIENT‐32 trial as the target population. The Markov model was constructed for economic evaluation of sintilimab combined with IBI305 as the first‐line treatment for patients with HCC from a Chinese payer perspective. This model has typically been used by researchers for pharmacoeconomic analyses of advanced and metastatic cancer treatment.^14^, ^15^ The model provided three health states: stable disease (SD), progressive disease (PD), and death. All patients were in an SD state in the initial stage. As the treatment progressed, the patient either moved to another state or stayed in this state. When the disease progressed, we assumed that the patients received an anti‐angiogenesis inhibitor (regorafenib) as standard second‐line treatment, as recommended by the 2021 Chinese Society of Clinical Oncology (CSCO) guidelines (version 2022) for HCC.^16^ Notably, once a patient enters the PD state, they cannot return to the SD state; they either remain in the PD state or are transferred to the death state during the subsequent cycle. The specific transitions of each state in the model are shown in Figure 1. The model was built and run using TreeAge Pro 2021 (Inc, Williamstown, MA, USA).

In the ORIENT‐32 clinical trial, the experimental group had not yet reached the median OS. However, immunotherapy has a delayed effect and may continue to exert its beneficial effects beyond the treatment period; therefore, it should be analyzed using long‐term data to avoid inaccuracies and uncertainties in the results. Hence, we set the cycle of the Markov model to 21 days and the time horizon to 10 years to simulate the entire life course of the patient.^17^ A half‐circle correction was conducted to accurately simulate the transfer process. This research was based on the perspective of Chinese payers, with a 5% discount on costs and utilities.^18^ We set the willingness‐to‐pay (WTP) threshold at three times China's GDP per capita in 2021 (US $38334). The key metric was the incremental cost–benefit ratios (ICERs), and secondary metrics included total costs, quality‐adjusted life years (QALYs), and life years (LYs). The research methods followed the Consolidated Health Economic Evaluation Reporting Standards (CHEERS) (Table S1).^19^

### 2.3 Clinical data input

The GetData Graph Digitizer (version 2.26) was used to extract the data points from the Kaplan–Meier curve of the ORIENT‐32 trial. R software was then used to reconstruct the extracted curve.^20^ We selected the best distribution from the exponential, Weibull, gamma, log‐normal, log‐logistic, and Gompertz distributions to fit the reconstructed individual patient data.^21^ According to the Akaike information criterion (AIC) and Bayesian information criterion (BIC), log‐normal distribution, and log‐logistic distributions had the best fit for survival data (Figure S1). Ishak et al. have reported that in the process of fitting the parameter distribution to the survival model, low AIC and BIC values provide objective criteria for the final selection of the distribution.^22^ The selection process for the distribution is presented in Table S2. The transition probability between the states of the Markov model was calculated using the method described by Liu et al.^23^ This method reasonably corrects the time‐dependent transition probability of a dynamic the Markov model.

### 2.4 Utility and cost estimates

As the health utility values for SD and PD status in the ORIENT‐32 trial were not publicly available, we used data from previously published HCC‐related studies as the health utility in SD and PD states (0.76 for SD and 0.68 for PD).^24^ To simplify the calculation, Grade 3 or 4 adverse events (AEs) with the highest incidence difference between the sintilimab plus IBI305 and sorafenib groups were selected. AEs included palmar‐plantar erythrodysesthesia syndrome, hypertension, and decreased platelet count, with incidence rates of 0, 14, 8% in the sintilimab plus IBI 305 group, and incidence rates of 12%, 6%, and 3% in the sorafenib group.

Costs were converted based on 2021 U.S. dollar exchange rates (USD 1.0 = CNY 6.34). We only considered the direct costs associated with medication, follow‐up treatment, disease management, laboratory tests, and major grade 3 or 4 AEs according to the ORIENT‐32 trial. We obtained the latest prices of the drugs involved in the study through the sales prices of local hospitals or by consulting local drug suppliers. For unresectable, advanced HCC, according to China's National Basic Medical Insurance (CNBMI) drug catalog (http://www.mohrss.gov.cn/xxgk2020/fdzdgknr/shbx_4216/gsbx/202112/t20211203_429397.html), sintilimab and sorafenib could be covered to partially reduce patient payments. The prices of the relevant drugs as costs both before and after health insurance coverage are presented in Table 1. Except for the AE‐related cost as a one‐time cost input model, the costs were calculated on a three‐week cycle. As some of the costs referred to previously published literature, we used the consumer price index (CPI) inflation calculator to adjust these costs to 2021 prices.^25^

**TABLE 1:** Key model input parameters.

Model variables	Baseline value	Lower limit	Upper limit	Distribution	Reference
Parameter survival distribution					
Log‐normal OS of S + B	mean log = 2.763	—	—	—	Model fitting
	sdlog = 0.971				
Log‐normal PFS of S + B	mean log =1.60	—	—	—	Model fitting
	sdlog =1.03				
Log‐logistic OS of S	shape = 1.68	—	—	—	Model fitting
	scale = 10.57				
Log‐normal PFS of S	mean log = 1.115	—	—	—	Model fitting
	sdlog = 0.781				
Risk for main adverse events					
Sintilimab + IBI305 therapy					
Palmar‐plantar erythrodysaesthesia syndrome	—	—	—	—	—
Hypertension	0.14	0.112	0.168	Beta	[11]
Decreased platelet count	0.08	0.064	0.096	Beta	[11]
Chemotherapy therapy					
Palmar‐plantar erythrodysaesthesia syndrome	0.12	0.096	0.144	Beta	[11]
Decreased neutrophil count	0.06	0.048	0.072	Beta	[11]
Hypertension	0.03	0.024	0.036	Beta	[11]
Health utility scores					
Utility of SD	0.76	0.608	0.912	Beta	[24]
Utility of PD	0.68	0.544	0.816	Beta	[24]
Main expenses, $/per cycle					
Sintilimab	340.848	272.848	408.848	Gamma	Local quotation
Sintilimab‐Medical insurance	119.2968	102.2544	136.3392	Gamma	Local quotation
IBI305	1644.5916	1315.5916	1973.5916	Gamma	Local quotation
Sorafenib	1259.244	1007.244	1511.244	Gamma	Local quotation
Sorafenib‐Medical insurance	629.622	539.676	719.568	Gamma	Local quotation
Regorafenib	800.2827	685.9566	914.6088	Gamma	Local quotation
Follow‐up	59.2	47.36	71.04	Gamma	[17]
Laboratory	157.5	126	189	Gamma	[40]
Administration	69.81	55.85	83.77	Gamma	[17]
Expenditures on main SAEs, $					
Palmar‐plantar erythrodysaesthesia syndrome	16.63	13.30	19.95	Gamma	[37]
Decreased platelet count	332.15	265.72	398.58	Gamma	[34]
Hypertension	155.56	124.45	186.68	Gamma	[34]
Disutility due to SAEs					
Palmar‐plantar erythrodysaesthesia syndrome	−0.15	−0.18	−0.12	Beta	[41]
Decreased platelet count	−0.146	−0.175	−0.117	Beta	[34]
Hypertension	−0.016	−0.019	−0.013	Beta	[42]

Abbreviations: OS, overall survival; PD, progressive disease; PFS, progression‐free survival; S + B, Sintilimab plus IBI305; S, Sorafenib; SAE, severe adverse event; SD, stable disease.

The drug dose was based on actual clinical trials. In the IBI305 plus sintilimab group, the patients received sintilimab (200 mg) and IBI305 (15 mg/kg) once every 3 weeks. In the control group, the patients received sorafenib (400 mg) twice daily. According to a report on the status of Chinese residents' nutrition and chronic diseases in 2020, the average weight of the adult Chinese population is 64.8 kg.^26^ However, considering the long progression of HCC, most patients are likely to be middle‐aged and older adults, and in the advanced stage of the disease, patients are likely to suffer from weight loss and other discomforts. Therefore, we assumed the average weight of patients to be 60 kg. The weight set would be used to calculate the drug dose per cycle for IBI305. Briefly, 29% (109/380) of patients in the sintilimab plus IBI305 group and 47% (89/191) in the sorafenib group received subsequent treatments. The regorafenib dose was based on the RESORCE trial, an RCT evaluating the efficacy of regorafenib in the treatment of HCC. These patients received 4 × 40 mg regorafenib orally once daily.^27^, ^28^ According to CNBMI drug catalog, regorafenib is considered as a treatment for HCC disease progression after the previous use of sorafenib, and we adopted the price of regorafenib under the medical insurance policy.^29^

### 2.5 Sensitivity analyses

A one‐way sensitivity analysis was conducted to explore the parameters that might affect the ICER and the extent to which they might do so. Each parameter was independently changed by assuming ±20% of the expected value to determine the evident influence on decision‐making. In the probabilistic sensitivity analysis (PSA), we selected appropriate distributions for the parameters relevant to the inclusion in the model; for example, costs (adverse effects of drugs and treatments) are gamma and risks (adverse effects) and health utility scores (SD, OS, and AE) are beta distributions. All parameters fluctuated between the 95% confidence interval (CI).^30^

## 3 RESULTS

### 3.1 Base‐case analysis

We analyzed both sintilimab and sorafenib without Medicare reimbursement and after Medicare coverage. According to our analysis, when sintilimab and sorafenib were not covered by Medicare, the incremental cost of sintilimab plus IBI305 ($44635.28, 1.3 QALYs) versus sorafenib ($27083.10, 0.97 QALYs) was $17552.17, QALYs was 0.33, and ICER value ($52817.89/QALY) was higher than WTP threshold ($38,334). When sintilimab and sorafenib were covered by Medicare, the incremental cost of sintilimab plus IBI305 ($42399.33, 1.3 QALYs) versus sorafenib ($24323.41, 0.97 QALYs) was $18075.93, QALYs was 0.33, and ICER value ($54393.97/QALY) was higher than WTP threshold ($38,334/QALY) (Table 2).

**TABLE 2:** Base‐case analysis results.

Analysis results	Strategies	
	Sintilimab plus IBI305	Sorafenib
Costs^a^ ($)	44635.28	27083.10
Incr costs^a^ ($)	17552.17	
Costs^b^ ($)	42399.33	24323.41
Incr costs^b^ ($)	18075.93	
QALYs	1.30	0.97
Incr QALYs	0.33	
ICER^a^ ($/QALY)	52817.89	
ICER^b^ ($/QALY)	54393.97	
WTP ($/QALY)	38,334	

Abbreviations: CPS≥10, PD‐L1 combined positive score of 10 or more; ICER, Incremental cost‐effectiveness ratio; Incr Cost, incremental cost; Incr QALYs, Incremental *quality*‐adjusted life‐years; PD‐L1, programmed cell death‐Ligand 1; QALYs, Quality‐adjusted life‐years.

^a^ Without Medicare reimbursement.

^b^ After Medicare coverage.

### 3.2 Sensitivity analyses

**FIGURE 2:**
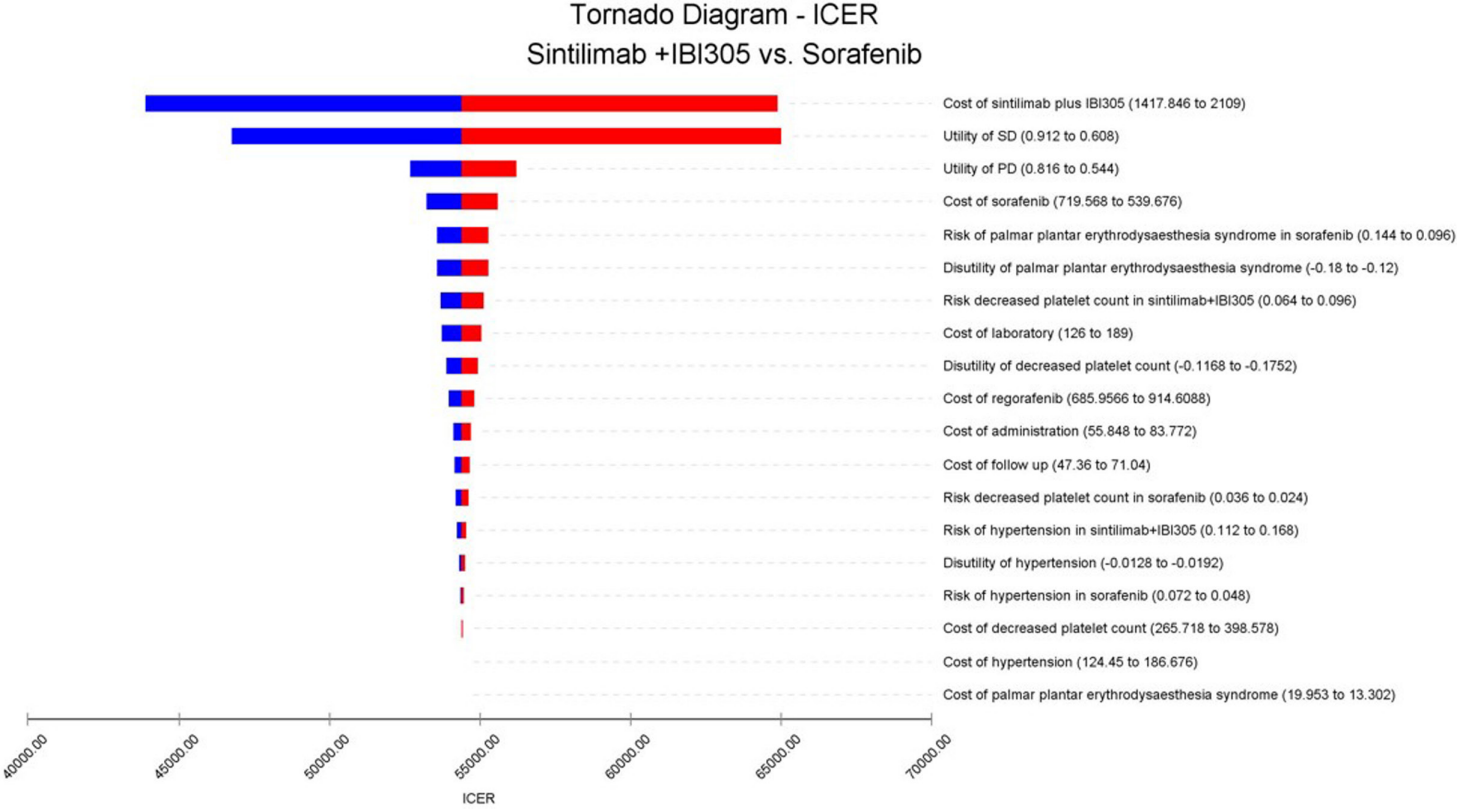
Tornado diagram for one‐way sensitivity analysis. The tornado diagram shows the effect of the model input parameters on the analysis results (ICER) as they vary over the range interval. The degree of influence of each parameter is presented from high to low. PD, progressive disease; SD, stable disease.

We further performed sensitivity analyses using the situation that both sintilimab and sorafenib are eligible for Medicare policy, because this situation is more realistic. Under the condition that the input model parameters changed by ±20%, the influence of each parameter change on the model output was explored by one‐way sensitivity analysis. The tornado diagram (Figure 2) demonstrated that the cost of sintilimab plus IBI305, the utility of SD, and PD were the main factors affecting the ICERs. Therefore, reducing the total cost of sintilimab plus IBI305 may increase the economic benefits of the experimental group. Additionally, when the total cost of sintilimab plus IBI305 was reduced by 31.9%, the ICER of sintilimab plus IBI305 would be equal to the WTP.

**FIGURE 3:**
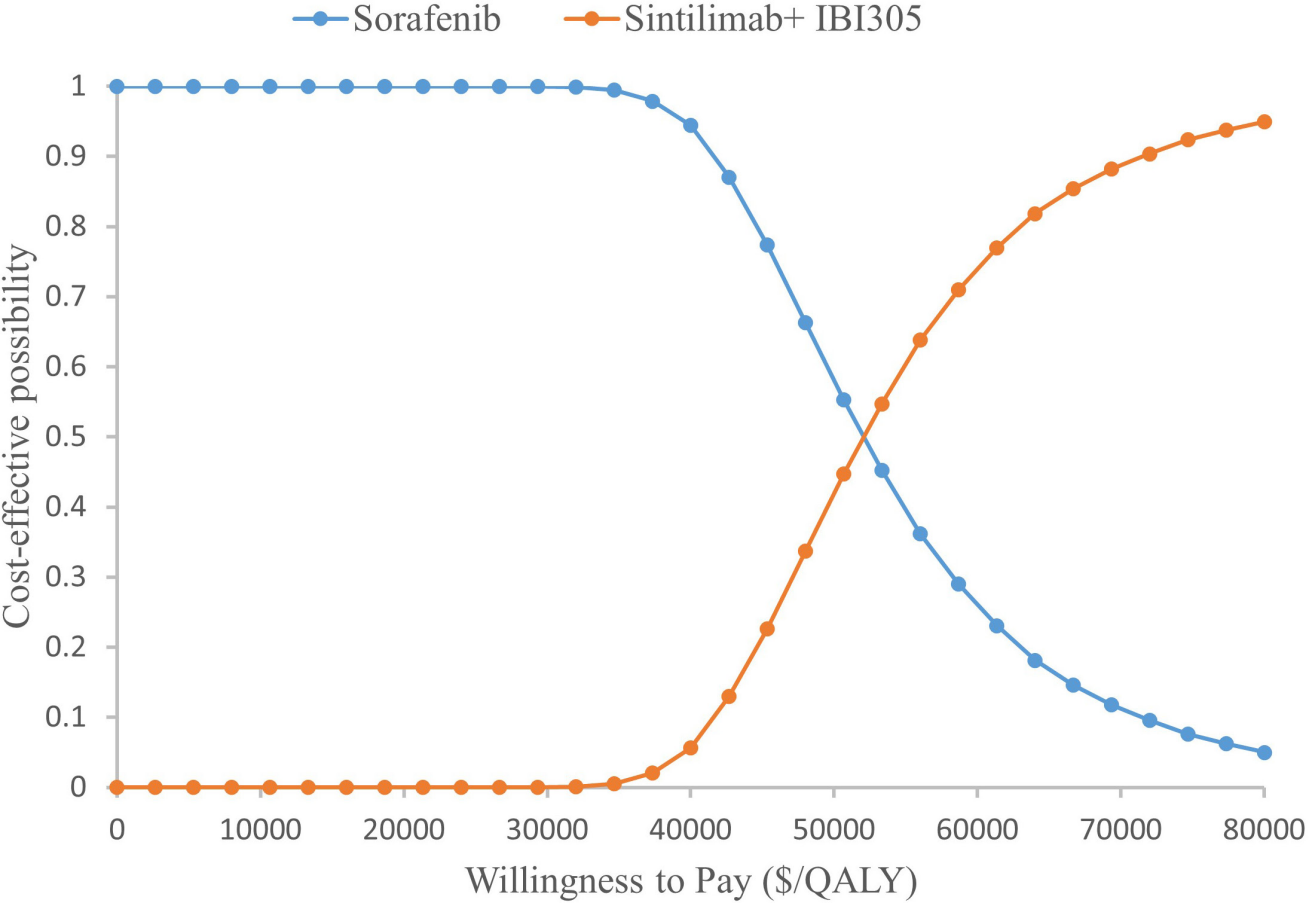
Cost‐effectiveness acceptability curves at different WTP thresholds. QALY, Quality adjusted life year; WTP, Willingness to pay.

PSA was performed to test the bias of the multiple model parameters on the analysis results when the multiple model parameters simultaneously changed. All model input parameters were varied within a given distribution, and 10,000 samples were obtained to generate the cost‐effectiveness acceptability curve (CEAC) and incremental cost‐effectiveness scatterplots in Figure 3 and Figure S2. Under the condition of WTP threshold of $38,334 (¥ 242,928) per QALY, CEAC showed that there was 1.28% probability that sintilimab plus IBI305 was cost‐effective compared with sorafenib over a range of fluctuations in all model parameters. Additionally, in the incremental cost–benefit scatter plot, all simulation points were below the WTP line.

## 4 DISCUSSION

This study is a pharmacoeconomic correlation analysis based on the ORIENT‐32 trial and the latest population data and drug prices in China. It is the first study to evaluate whether sintilimab plus IBI305 is more cost‐effective than sorafenib as a first‐line treatment for patients with HCC in China.

The IMBRAVE150 and ORIENT‐32 trials have verified the success of immunotherapy combined with antiangiogenic drugs.^10^, ^11^ In particular, the excellent efficacy of ORIENT‐32 in the Chinese population has established a milestone in the treatment of HCC. Before 2017, sorafenib was considered as having the best clinical evidence for the treatment of unresectable HCC.^31^ Owing to the ORIENT‐32 trials showing the sintilimab plus IBI305 has considerable efficacy in patients with HCC, the first‐line standard treatment options in China may be changed. However, owing to high drug prices and treatment costs, patients are typically forced to abandon the best treatment plan. Therefore, clinicians must combine the dual benefits of clinical efficacy and costs to make personalized clinical decisions. To avoid wasting medical resources, a cost‐effectiveness analysis should be conducted.

The country's per capita GDP is an important univariate predictor of willingness to bear the burden of cancer treatment.^32^ Our economic assessment revealed that with a WTP threshold of $38,334 (¥ 242980.059)/QALY, sintilimab plus IBI305 may not be a cost‐effective option for patients with HCC, even if it offers the patients more survival benefits. In addition, the cost of IBI305 and sintilimab plus IBI305 was the most sensitive to the analysis results. We performed 10,000 simulations in the model, and the results showed that the probability of sintilimab plus IBI305 being cost‐effective was 8.7%.

In the ORIENT‐32 clinical trials, different follow‐up treatments (local regional therapy, radiation therapy, surgery, and PD‐1 inhibitors) were selected according to the actual state of patients upon disease progress. A large proportion (21%) of patients received PD‐1 inhibitors in the sorafenib group, whereas only 2% of patients received PD‐(L) 1 inhibitors in sintilimab plus IBI305 group, which would greatly increase the treatment cost of patients with PD status in the sorafenib group, leading to the overestimation of the economic benefits of sintilimab plus IBI305. Therefore, we hypothesized that both groups of patients received donafenib at the time of disease progression to avoid this bias, although its efficiency was different from that of patients with actual PD status. In addition, as the control group included patients receiving sorafenib, the price of the sorafenib largely determines cost‐effectiveness. The lower the price of sorafenib, the lower the ICERs of the experimental group. For the cost of sorafenib, we used the actual price charged by a local hospital, which is approximately $59.964 per day and $1259.244 per 3 weeks. In the study by Cai et al., this cost was $2328.05 per 3 weeks.^33^ There have been some economic studies on sorafenib for HCC; however, they primarily compared with chemotherapy regimens such as FOLFOX4.^34^ Chemotherapy is considerably less expensive than immunotherapy in most cases; therefore, it is more cost‐effective. Another study revealed that the ICER for atezolizumab and bevacizumab combination therapy was higher than the WTP threshold for US payers compared to sorafenib monotherapy.^35^ Therefore, PD‐1 inhibitors may be difficult to recommend as cost‐effective options in unresectable HCC unless PD‐1 inhibitors undergo a further price reduction.

There are a few cost‐effectiveness studies on HCC. Kobayashi et al. revealed that lenvatinib is a cost‐effective alternative to sorafenib in Japan.^36^ This treatment option is equally cost‐effective in other economically developed countries.^33^, ^37^ Based on the IMBRAVE150 clinical trial, Wen et al. conducted an economic evaluation of atezolizumab plus bevacizumab treatment regimen in China.^38^ The ICER obtained was $145546.21 per QALY, which clearly exceeded the WTP threshold. Compared with our analysis results, atezolizumab plus bevacizumab has a higher cost and ICER, indicating that it is inferior to the sintilimab plus IBI305 treatment regimen. However, there are some major differences. First, the simulated data of our study and that of Wen et al. are based on different clinical trials; the treatment population of ORIENT‐32 is from China, whereas that of IMBRAVE150 is from multiple centers worldwide. Although the IMBRAVE150 study included subgroup analysis data of some Asian populations, ORIENT‐32 is more accurate to the real curative effect in the Chinese population. Additionally, in our study, the drug prices are determined by China's National Basic Medical Insurance, which will be implemented starting January 1, 2022. Sintilimab plus IBI305 and sorafenib are eligible for Medicare reimbursement for patients with HCC, which has led to a significant reduction in treatment costs. Second, although the two studies used the same utility for PFS and PD status, Wen et al. did not consider the loss of utility caused by AEs, which was fully considered in our model. As the incidence of AEs was higher with sintilimab plus IBI305 than with sorafenib, the sintilimab plus IBI305 group had lower utility. Third, the transition probability in the Markov model of Wen et al. is a fixed value; however, we obtained the dynamic time‐dependent transition probability by fitting the mathematical function of public km data, which is more in line with the state transition process of patients in a real situation. Therefore, the difference in cost‐effectiveness between the two treatment schemes should be carefully considered and requires further research. In addition, although the price of sintilimab plus IBI305 is lower, sintilimab plus IBI305 has incurred high treatment costs in the treatment process as well as atezolizumab plus bevacizumab, which is not a cost‐effective choice in China.

Sintilimab has been included in China's National Basic Medical Insurance List, which is encouraging. However, it is currently limited to patients with refractory classical Hodgkin's lymphoma who have failed second‐line chemotherapy. The combination of sintilimab plus IBI305 is still a heavy economic burden for patients with HCC. The asymmetry between incremental treatment value and the incremental cost of combination therapy is a huge challenge to the affordability of payers, causing combination therapies for ICIs to be rejected or restricted in many medical institutions.^39^ Therefore, reducing the price of sintilimab and IBI305 is an effective and feasible strategy that can benefit patients economically. In the past, the National Medical Security Administration negotiated to include sorafenib in the medical insurance catalog at a lower price, bringing new hope to patients with HCC.

### 4.1 Limitations

Our study had some limitations. First, patients simulated by our model were obtained from the ORIENT‐32 trial. The translation of clinical trial results into clinical practice is uncertain. For example, trial participants tended to cooperate more actively with treatment than real‐world patients, which may overestimate the effectiveness of sintilimab plus IBI305 therapy. Second, the follow‐up period of the ORIENT‐32 trial was short. The sintilimab plus IBI305 group did not reach the median OS time, and its long‐term efficacy should be updated by subsequent follow‐ups. Survival outside the patient model in this study was predicted by the parameter distribution; therefore, this will inevitably be biased with real‐world patient data. Third, the utilities we used for each health state were extracted from previous studies, and the true health utilities of patients with HCC are difficult to obtain. Therefore, we performed sensitivity analyses to reduce uncertainty. The incidence of AEs with sintilimab plus IBI305 therapy was greater than with sorafenib monotherapy, resulting in greater loss of utility. However, the quality‐of‐life assessment published in the ORIENT‐32 trial reported that patients treated with sintilimab plus IBI305 received more health quality benefits; therefore, the incremental utility of the actual sintilimab plus IBI305 group may be higher. Fourth, the patient's subsequent treatment plan may have potentially affected the outcome. For example, 21% of patients received ICIs in the sorafenib group, whereas only 2% received ICIs in the sintilimab plus IBI305 group, which exaggerated the efficacy of the sorafenib group.

## 5 CONCLUSIONS

Overall, regardless of whether the price of sintilimab plus IBI305 and sorafenib is covered by Medicare, sintilimab plus IBI305 is unlikely to be cost‐effective as a first‐line treatment for patients with unresectable HCC in China, and further reductions in the price of sintilimab and IBI305 are required.

## AUTHOR CONTRIBUTIONS

**Zhuo‐miao Ye:** Conceptualization (equal); data curation (equal); formal analysis (equal); methodology (equal); resources (equal); software (equal); supervision (equal); validation (equal); visualization (equal); writing – original draft (equal); writing – review and editing (equal). **Zhe Xu:** Conceptualization (equal); data curation (equal); formal analysis (equal); methodology (equal); resources (equal); software (equal); validation (equal); visualization (equal); writing – original draft (equal); writing – review and editing (equal). **Yu‐kai Tang:** Writing – original draft (equal); writing – review and editing (equal). **Dong‐feng Deng:** Writing – original draft (equal). **Qin Zhou:** Project administration (equal); supervision (equal). **Man Fang:** Supervision (equal). **Ying‐ying Zhang:** Funding acquisition (equal); investigation (equal); project administration (equal);writing – review and editing (equal). **Xiang‐Ping Li:** Funding acquisition (equal); investigation (equal); methodology (equal); project administration (equal); supervision (equal); writing – review and editing (equal).

## FUNDING INFORMATION

This study was funded by Natural Science Foundation of Hunan Province (No. 2022JJ30985).

## CONFLICT OF INTEREST STATEMENT

The authors declare no conflict of interest.

## ETHICS STATEMENT

Not applicable.

## INFORMED CONSENT STATEMENT

Not applicable.

## Supporting information


Figure S1.

Figure S2.

Table S1.

Table S2.

## Data Availability

The data supporting the findings of this study are available from the corresponding author upon reasonable request.
