# Supplementary figures and images for: Cost‐effectiveness analysis of sintilimab plus IBI305 versus sorafenib for unresectable hepatic cell carcinoma in China
Source: Cancer Med. 2023 Jul 11;12(14):14871–80. doi: 10.1002/cam4.5724 (PMC10417160; doi:10.1002/cam4.5724)

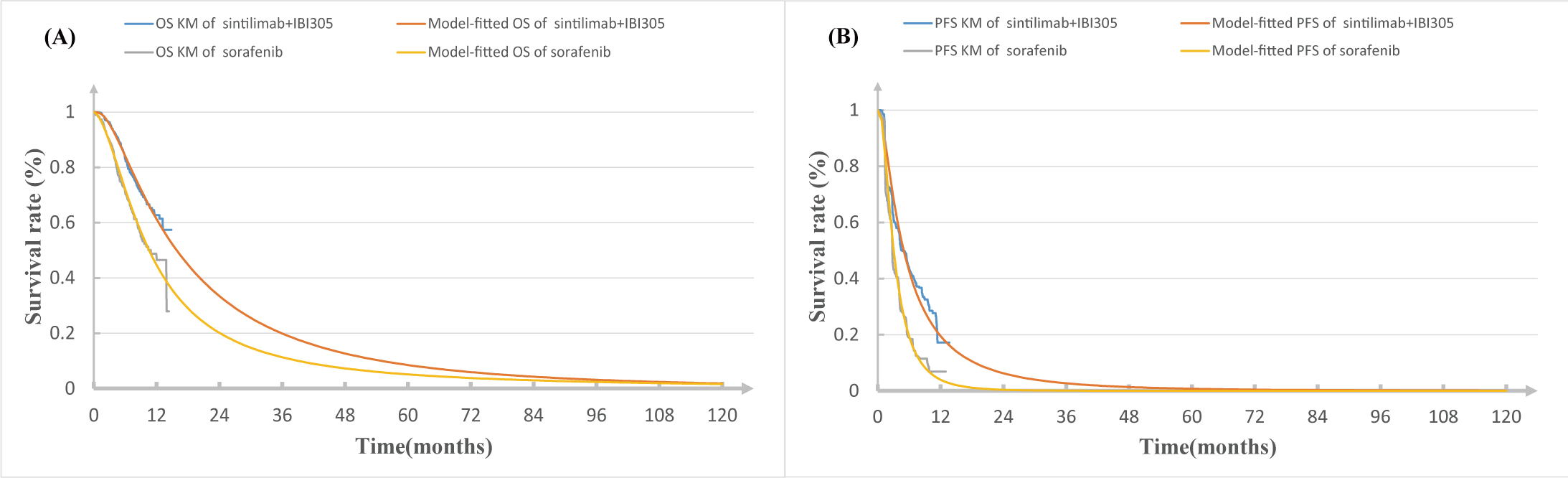

Supplement: Supplementary file 1 — Figure S1. [file CAM4-12-14871-s003.jpg]

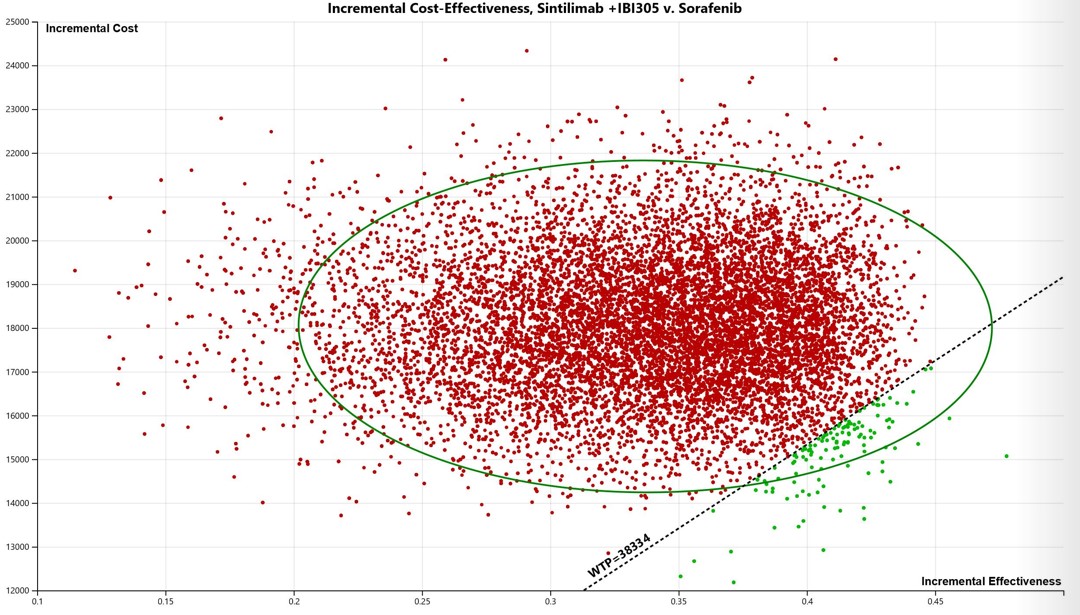

Supplement: Supplementary file 2 — Figure S2. [file CAM4-12-14871-s004.jpg]
