# Supplementary material for: Cost‐effectiveness analysis of sintilimab plus IBI305 versus sorafenib for unresectable hepatic cell carcinoma in China
Source: Cancer Med. 2023 Jul 11;12(14):14871–80. doi: 10.1002/cam4.5724 (PMC10417160; doi:10.1002/cam4.5724)
Supplement: Supplementary file 4 — Table S2. [file CAM4-12-14871-s001.docx]

Table2.AIC and BIC scores of fitted distribution in sintilimab plus IBI305 group and sorafenib group

| Distribution | OS of sintilimab + IBI305 | | | OS of sorafenib | | PFS of sintilimab + IBI305 | | PFS of sorafenib | |
| --- | --- | --- | --- | --- | --- | --- | --- | --- | --- |
|  | AIC | BIC | AIC | | BIC | AIC | BIC | AIC | BIC |
| Exponential | 1047.006 | 1054.886 | 672.831 | | 679.335 | 1509.139 | 1517.019 | 704.309 | 710.814 |
| Gamma | 1013.847 | 1025.668 | 662.151 | | 671.908 | 1496.634 | 1508.455 | 671.458 | 681.215 |
| Weibull | 1017.286 | 1029.106 | 663.135 | | 672.892 | 1503.089 | 1514.910 | 682.313 | 692.070 |
| Log-normal | 1008.982 | 1020.802 | 664.440 | | 674.197 | 1456.598 | 1468.418 | 653.741 | 663.498 |
| Log-logistic | 1012.815 | 1024.636 | 661.056 | | 670.813 | 1476.645 | 1488.466 | 654.482 | 664.239 |
| Gompertz | 1032.600 | 1044.420 | 668.798 | | 678.554 | 1511.138 | 1522.959 | 702.418 | 712.175 |

Abbreviation: OS=Overall survival; PFS= Progression-free survival; AIC= Akaike information criterion; BIC= Bayesian information criterion.
